# Effects of Total Pancreatectomy on Survival of Patients With Pancreatic Ductal Adenocarcinoma: A Population-Based Study

**DOI:** 10.3389/fsurg.2021.804785

**Published:** 2021-12-09

**Authors:** Weiwei Shao, Zhenhua Lu, Jingyong Xu, Xiaolei Shi, Tianhua Tan, Cheng Xing, Jinghai Song

**Affiliations:** ^1^Department of General Surgery, Beijing Hospital, National Center of Gerontology, Institute of Geriatric Medicine, Chinese Academy of Medical Sciences, Beijing, China; ^2^Graduate School of Peking Union Medical College, Chinese Academy of Medical Sciences, Beijing, China

**Keywords:** pancreatic ductal adenocarcinoma, total pancreatectomy, propensity score matching, prognosis, nomogram, SEER

## Abstract

**Background:** Total pancreatectomy (TP) seems to be experiencing a renaissance in recent years. In this study, we aimed to determine the long-term survival of pancreatic ductal adenocarcinoma (PDAC) patients who underwent TP by comparing with pancreaticoduodenectomy (PD), and formulate a nomogram to predict overall survival (OS) for PDAC individuals following TP.

**Methods:** Patients who were diagnosed with PDAC and received PD (*n* = 5,619) or TP (*n* = 1,248) between 2004 and 2015 were selected from the Surveillance, Epidemiology, and End Results (SEER) database. OS and cancer-specific survival (CSS) of the PD and TP groups were compared using Kaplan-Meier method and log-rank test. Furthermore, Patients receiving TP were randomly divided into the training and validation cohorts. Univariate and multivariate Cox regression were applied to identify the independent factors affecting OS to construct the nomogram. The performance of the nomogram was measured according to concordance index (C-index), calibration plots, and decision curve analysis (DCA).

**Results:** There were no significant differences in OS and CSS between TP and PD groups. Age, differentiation, AJCC T stage, radiotherapy, chemotherapy, and lymph node ratio (LNR) were identified as independent prognostic indicators to construct the nomogram. The C-indexes were 0.67 and 0.69 in the training and validation cohorts, while 0.59 and 0.60 of the American Joint Committee on Cancer (AJCC) tumor-node-metastasis (TNM) staging system. The calibration curves showed good uniformity between the nomogram prediction and actual observation. DCA curves indicated the nomogram was preferable to the AJCC staging system in terms of the clinical utility. A new risk stratification system was constructed which could distinguish patients with different survival risks.

**Conclusions:** For PDAC patients following TP, the OS and CSS are similar to those who following PD. We developed a practical nomogram to predict the prognosis of PDAC patients treated with TP, which showed superiority over the conventional AJCC staging system.

## Introduction

Pancreatic cancer remains a devastating disease. According to the latest reports, pancreatic cancer ranks the fourth in the tumor-related death in the United States in 2020, with the 5-year survival rate of only 9% (1). Pancreatic ductal adenocarcinoma (PDAC) is the most common histopathological type and has almost been synonymous with pancreatic cancer (2). Surgical resection is the only known curative method. As we all know, there are three main surgical approaches for PDAC generally based on the location of the lesion, pancreaticoduodenectomy (PD), distal pancreatectomy (DP) and total pancreatectomy (TP). While PD and DP have been common surgical approaches with confirmed short- and long-term outcomes (3, 4), the role of TP in the treatment of PDAC remains controversial.

TP was first performed by Rockey in 1943 for PDAC, but the patient died of severe bile duct leakage 15 days later (5). TP, a resection of the entire gland, was considered as a more radical surgical method which can effectively avoid potential postoperative pancreatic fistula (POPF) and minimize the risk of tumor recurrence in early period (6). However, TP was then shown to lead to higher perioperative morbidity and mortality than PD (7). Additionally, TP strongly influences patients' metabolic function and postoperative quality of life (QoL) due to permanent pancreatic endo-exocrine insufficiency (8). Despite these adverse effects, TP is still required in some cases to achieve a negative resection margin and complete clearance (9). Several studies have compared perioperative morbidity and mortality between TP and PD, but data on long-term survival benefit between the two surgical methods are still minimal and even controversial (7, 10–14). Since most previous reports were single-center and small sample size investigations, further exploration concerning the long-term survival benefit of TP is needed.

PDAC is heterogeneous among individuals regarding survival, so a practical and personalized prognostic tool that can predict the survival probability is necessary and helpful. The American Joint Committee on Cancer (AJCC) tumor-node-metastasis (TNM) staging system has been commonly used for prognostic prediction after surgery (15). However, the TNM staging system includes only lesion size, positive lymph nodes on pathological examination, and presence of distant metastasis. Other factors such as age, sex, serum carbohydrate antigen 19-9 (CA 19-9), tumor differentiation, lymph node ratio (LNR), and even marital status are also considered to be related to the prognosis of PDAC (16–19).

Nomogram models are novel, simple and convenient mathematical tools for prognostic prediction in clinical practice; it incorporates important demographic and clinicopathological characteristics to forecast individual prognosis more precisely (20). The Surveillance, Epidemiology, and End Results (SEER) in the United States is a long-established and open access database providing population-based statistics and information on various cancers. In this study, by using data of SEER, we were aiming to probe the long-term survival of PDAC patients who underwent TP by comparing them with those who underwent PD, and also to formulate a prognostic nomogram to better predict overall survival (OS) for PDAC individuals following TP.

## Materials and Methods

### Patients Selection and Data Extraction

This retrospective study focused on patients who were diagnosed with PDAC and treated with PD and TP from 2004 to 2015, with the last follow-up in November 2018. All the subjects were extracted from the SEER database (SEER^*^Stat 8.3.9). Patients diagnosed with PDAC were selected using the site codes (C25.0-25.9) and histology codes (8140 and 8500) of the International Classification of Disease for Oncology, 3rd edition (ICD-O-3). Surgery codes of PD and TP were 37 and (40, 60), respectively. The exclusion criteria were as follows: (1) The carcinoma had metastasized; (2) incomplete or absence information about survival time, overall life status, cause of death, or other characteristics; (3) non-primary tumor; (4) age at diagnosed < 18 years old. Demographic and clinicopathological data, including age, sex, race, marital status, surgical methods, tumor location, size, differentiation, T and N stage of AJCC system, LNR, radiotherapy, chemotherapy, survival time, death reasons, and living status were extracted from the dataset.

The value of LNR was defined as the ratio of the number of positive lymph nodes to the total number of examined nodes. Overall survival (OS) was defined as the time from diagnosis to death due to any cause. Cancer-specific survival (CSS) was defined as the duration from diagnosis to death related to PDAC. The 6th or 7th AJCC TNM stage was transformed into the 8th AJCC TNM stage.

### Survival Analysis of TP and PD

We divided the patients according to their surgical treatment. Propensity score matching (PSM) analysis was applied to adjust for confounders and reduce the effect of selection bias (21). The X-tile program (Yale University, New Haven, CT, USA) was used to acquire the best cutoff values of age, tumor size, year and LNR (22). The two groups were matched in a 1:1 ratio using the nearest-neighbor method with a caliper of 0.01. The Kaplan-Meier method and log-rank test were used for the survival analysis.

### Prognostic Nomogram for TP

We randomly divided the patients who underwent TP into the training and validation cohorts at a ratio of 7:3. The nomogram for TP survival prediction was constructed based on the training cohort. Univariate and multivariate Cox proportional-hazards models were used to determine the prognostic factors. Factors in the nomogram for 1-, 3-, and 5-year OS prediction were based on the results of the multivariate Cox regression analysis. The nomogram model was validated by the two cohorts. The discriminative capacity was evaluated by the concordance index (C-index) (23). The C-index ranged from 0.5 to 1, with larger values indicating better prediction accuracy. Calibration was evaluated by drawing calibration curves to investigate the consistency between the predicted probabilities and actual survival outcomes (24). The predictive ability of the nomogram was evaluated using 1,000 bootstrap resamples. Decision curve analysis (DCA), a novel algorithm, was performed to assess the clinical value of the nomogram by quantifying net benefit at different threshold probabilities (25). Moreover, according to the cutoff values calculated by X-tile, the overall scores calculated from the nomogram were classified into three groups, low-risk, intermediate-risk, and high-risk groups. Kaplan-Meier analysis and log-rank test were applied to compare the OS of different groups, testing whether the nomogram model could distinguish patients with different survival risks.

### Statistical Analysis

Continuous variables were shown as medians and interquartile range (IQR), while categorical variables were displayed as numbers and percentages. Features of Cox regression were presented as hazard ratio (HR) and corresponding 95% confidence intervals (CI). A student's *t*-test or Mann-Whitney *U*-test was used for continuous variables and chi-square test for categorical variables. Two-tailed *P*-values < 0.05 were considered statistically significant. Statistical analyses were conducted using R software (version 4.0.1 http://www.r-project.org).

## Results

### Characteristics of the Included Patients

**Figure 1:**
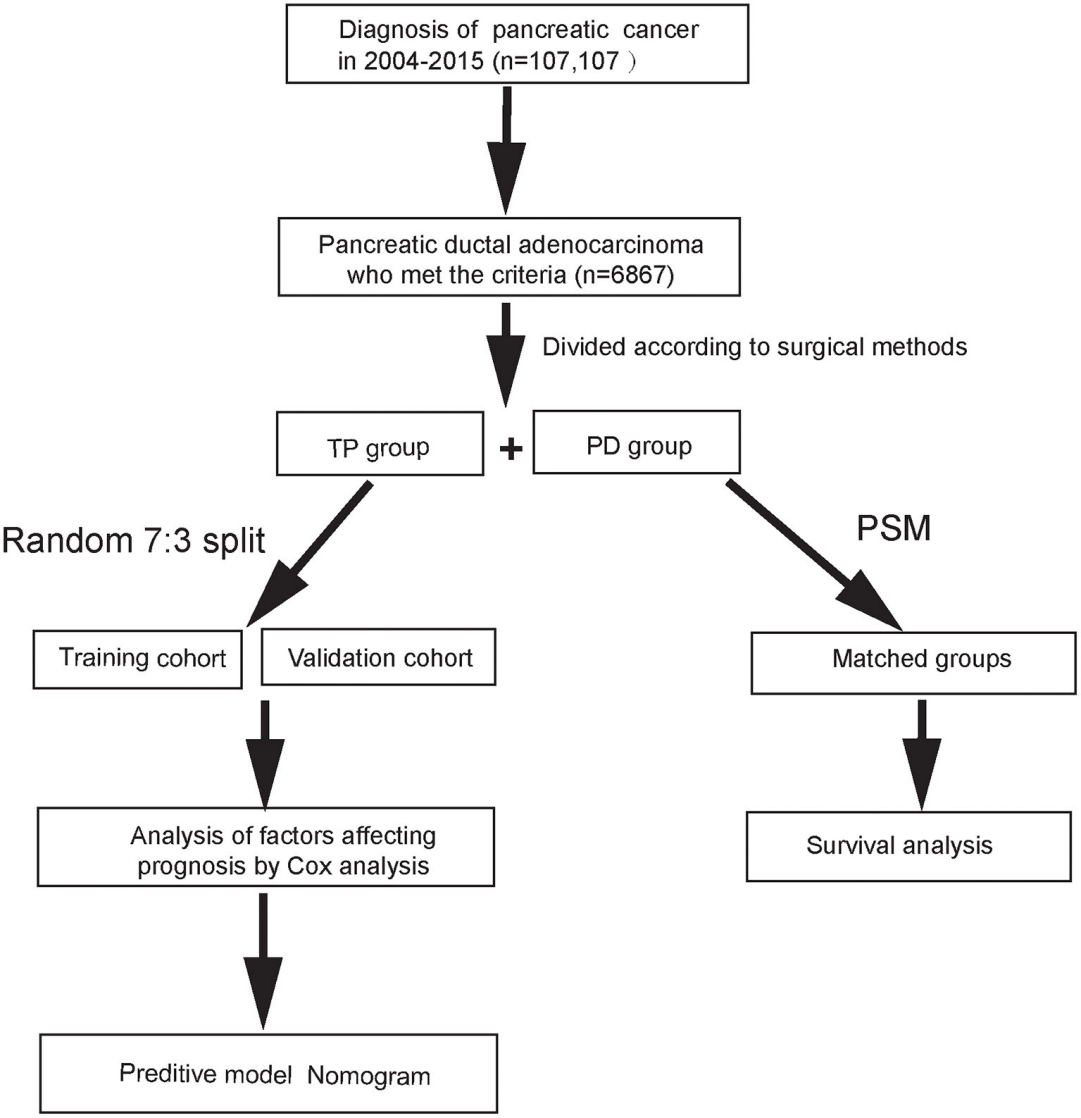
Simple flow diagram of the study.

A total of 6,867 patients with PDAC were screened from the SEER database from 2004 to 2015. Among these patients, 5,619 underwent PD and 1,248 received TP. According to the X-tile program, age was divided into <57 years old, 57–76 years old, and 77 years old or more; tumor size into <25, 25–33, and 34 mm or more; LNR into <0.07, 0.07–0.23, and 0.24 or more (Supplementary Figures S1A–C). Features of the patients were displayed in Table 1. After a 1:1 PSM, all baseline data were comparable between the two matched cohorts containing 1,248 pairs. Figure 1 showed the research process of this study.

**Table 1:** Demographic and clinicopathological characteristics of the patients before and after PSM.

**Characteristics**	**Original cohort (***n*** = 6,867)**			**Matched cohort (*****n*** **= 2,496)**		
	**PD (*n* = 5,619) *n* (%)**	**TP (*n* = 1,248) *n* (%)**	***P*-value**	**PD (*n* = 1,248) *n* (%)**	**TP (*n* = 1,248) *n* (%)**	***P*-value**
Age (year)			0.81			0.34
≤ 56	1,137 (20.2)	255 (20.4)		237 (19.0)	255 (20.4)	
57–76	3,636 (64.7)	797 (63.9)		832 (66.7)	797 (63.9)	
≥77	846 (15.1)	196 (15.7)		179 (14.3)	196 (15.7)	
Gender			0.71			0.90
Female	2,877 (51.2)	631 (50.6)		635 (50.9)	631 (50.6)	
Male	2,742 (48.8)	617 (49.4)		613 (49.1)	617 (49.4)	
Marital status			0.82			1.00
Married	3,534 (62.9)	780 (62.5)		779 (62.4)	780 (62.5)	
Other status	2,085 (37.1)	468 (37.5)		469 (37.6)	468 (37.5)	
Race			0.20			0.74
White	4,601 (81.9)	999 (80.0)		1,006 (80.6)	999 (80.0)	
Black	559 (9.9)	129 (10.3)		114 (9.1)	129 (10.3)	
Asian	426 (7.6)	115 (9.2)		122 (9.8)	115 (9.2)	
Other	33 (0.6)	5 (0.4)		6 (0.5)	5 (0.4)	
Tumor location			**<0.001**			1.00
Head	5,086 (90.5)	970 (77.7)		970 (77.7)	970 (77.7)	
Other	533 (9.5)	278 (22.3)		278 (22.3)	278 (22.3)	
Differentiation			0.55			0.27
Well	560 (10.0)	113 (9.1)		115 (9.2)	113 (9.1)	
Moderate	2,953 (52.6)	659 (52.8)		662 (53.0)	659 (52.8)	
Poor	2,057 (36.6)	461 (36.9)		465 (37.3)	461 (36.9)	
Undifferentiated	49 (0.9)	15 (1.2)		6 (0.5)	15 (1.2)	
Tumor size (mm)			0.36			0.78
≤ 24	1,426 (25.4)	297 (23.8)		283 (22.7)	297 (23.8)	
25–33	1,763 (31.4)	386 (30.9)		386 (30.9)	386 (30.9)	
≥34	2,430 (43.2)	565 (45.3)		579 (46.4)	565 (45.3)	
8th AJCC T stage			0.20			0.62
T1	598 (10.6)	129 (10.3)		133 (10.7)	129 (10.3)	
T2	3,296 (58.7)	697 (55.8)		679 (54.4)	697 (55.8)	
T3	1,502 (26.7)	367 (29.4)		390 (31.2)	367 (29.4)	
T4	223 (4.0)	55 (4.4)		46 (3.7)	55 (4.4)	
8th AJCC *N* stage			0.72			0.54
*N*0	1,698 (30.2)	391 (31.3)		399 (32.0)	391 (31.3)	
*N*1	2,372 (42.2)	522 (41.8)		496 (39.7)	522 (41.8)	
*N*2	1,549 (27.6)	335 (26.8)		353 (28.3)	335 (26.8)	
Chemotherapy			**<0.001**			0.87
No	1,574 (28.0)	413 (33.1)		418 (33.5)	413 (33.1)	
Yes	4,045 (72.0)	835 (66.9)		830 (66.5)	835 (66.9)	
Radiotherapy			**0.04**			0.41
No	3,316 (59.0)	777 (62.3)		798 (63.9)	777 (62.3)	
Yes	2,303 (41.0)	471 (37.7)		450 (36.1)	471 (37.7)	
LNR			0.23			0.65
≤0.06	2,218 (39.5)	525 (42.1)		511 (40.9)	525 (42.1)	
0.07–0.23	1,807 (32.2)	379 (30.4)		372 (29.8)	379 (30.4)	
≥0.24	1,594 (28.4)	344 (27.6)		365 (29.2)	344 (27.6)	

*AJCC, American Joint Committee on Cancer; LNR, lymph node ratio; PD, pancreaticoduodenectomy; PSM, propensity score matching; TP, total pancreatectomy. Bold values meant statistically significant*.

### Treatment Effects of TP vs. PD on Survival

**Figure 2:**
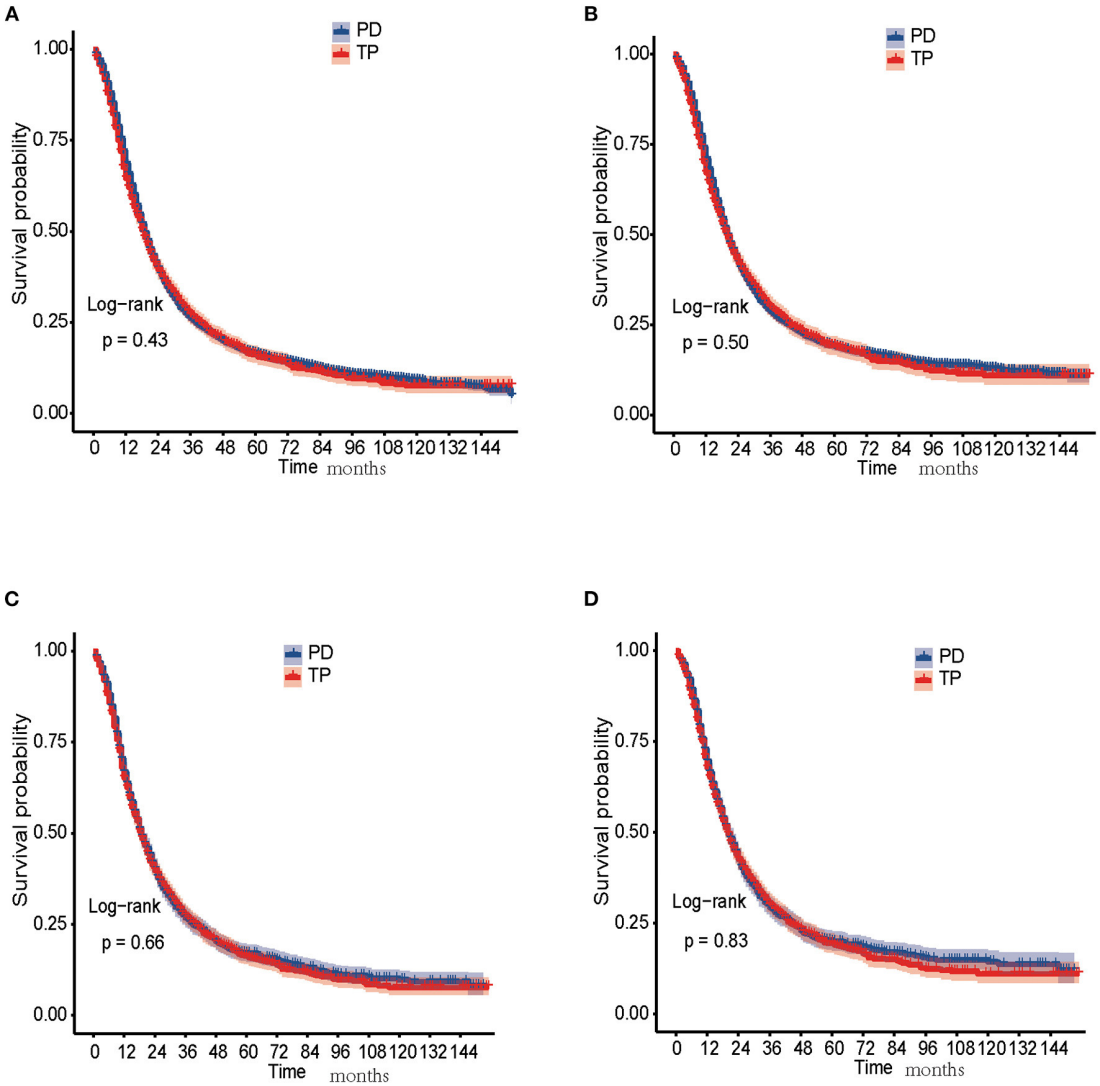
Survival analysis of PDAC patients treated with PD and TP. **(A)** OS curves of PD and TP groups before PSM; **(B)** CSS curves of PD and TP groups before PSM; **(C)** OS curves of PD and TP groups after PSM; **(D)** CSS curves of PD and TP groups after PSM. CSS, cancer-specific survival; OS, overall survival; PD, pancreaticoduodenectomy; PDAC, pancreatic ductal adenocarcinoma; PSM, propensity score matching; TP, total pancreatectomy.

In the unmatched cohort, the 1-, 3-, and 5-year OS rates in the PD group were 68.5, 26.1, and 16.4%, while 64.5, 27.0, and 16.1% in the TP group, respectively. The 1-, 3-, and 5-year CSS rates in the PD group were 70.8, 28.6, and 19.1%, while 67.1, 29.8, and 19.1% in the TP group, respectively. The Kaplan-Meier analysis and log-rank test showed that both OS (*P* = 0.43) and CSS (*P* = 0.50) in the TP and PD groups were similar and no significant differences were found (Figures 2A,B).

In the matched cohort, the 1-, 3-, and 5-year OS rates in the PD group were 66.1, 26.2, and 17.0%, while 64.5, 27.0, and 16.1% in the TP group, respectively. The 1-, 3-, and 5-year CSS rates in the PD group were 68.7, 28.8, and 19.6%, while 67.1, 29.8, and 19.1% in the TP group, respectively. No significant differences were detected in both OS (*P* = 0.66) and CSS (*P* = 0.83) between the two groups (Figures 2C,D).

### Analysis of Variables and Affecting OS Among TP Patients

Cox regression analysis were operated in the training cohort to determine the prognostic factors for PDAC patients after TP. Univariate analysis identified that age, tumor size, differentiation, 8th AJCC T and N stage, radiotherapy, chemotherapy, and LNR were significantly associated with OS. Additionally, multivariate analysis revealed age, differentiation, 8th AJCC T stage, radiotherapy, chemotherapy, and LNR were independent prognostic indicators (Table 2).

**Table 2:** Univariate and multivariate Cox analysis of variables affecting OS for PDAC patients following TP.

**Characteristics**	**Univariate analysis**			**Multivariate analysis**		
	**HR**	**95% CI**	***P*-value**	**HR**	**95% CI**	***P*-value**
**Age (year)**						
≤ 56	Reference			Reference		
57–76	1.15	0.95–1.39	0.15	0.96	0.96–1.42	0.12
≥77	1.56	1.22–1.99	**<0.001**	1.44	1.12–1.86	**0.005**
**Gender**						
Female	Reference					
Male	1.08	0.93–1.25	0.34			
**Marital status**						
Married	Reference					
Other status	1.14	0.98–1.32	0.10			
**Race**						
White	Reference					
Black	0.89	0.69–1.13	0.34			
Asian	0.93	0.71–1.22	0.59			
Other	4.43	0.62–31.62	0.14			
**Tumor location**						
Head	Reference					
Other	0.93	0.77–1.12	0.45			
**Tumor differentiation**						
Well	Reference			Reference		
Moderate	1.70	1.25–2.31	**0.001**	1.50	1.10–2.05	**0.01**
Poor	2.65	1.94–3.63	**<0.001**	2.24	1.63–3.09	**<0.001**
Undifferentiated	2.27	1.17–4.38	**0.02**	1.69	0.86–3.33	0.13
**Tumor size (mm)**						
≤ 24	Reference			Reference		
25–33	1.87	1.51–2.30	**<0.001**	1.27	0.99–1.63	0.06
≥34	1.78	1.47–2.17	**<0.001**	1.19	0.89–1.58	0.24
**8th AJCC T stage**						
T1	Reference			Reference		
T2	2.31	1.74–3.07	**<0.001**	1.70	1.21–2.38	**0.002**
T3	2.51	1.87–3.37	**<0.001**	1.93	1.28–2.91	**0.002**
T4	2.93	1.91–4.49	**<0.001**	2.73	1.67–4.46	**<0.001**
**8th AJCC** ***N*** **stage**						
*N*0	Reference			Reference		
*N*1	1.61	1.34–1.93	**<0.001**	1.20	0.90–1.60	0.22
*N*2	2.12	1.73–2.58	**<0.001**	1.19	0.83–1.70	0.35
**Chemotherapy**						
No	Reference			Reference		
Yes	0.66	0.56–0.77	**<0.001**	0.57	0.47–0.69	**<0.001**
**Radiotherapy**						
No	Reference			Reference		
Yes	0.79	0.67–0.92	**0.002**	0.83	0.70–0.99	**0.04**
**LNR**						
≤0.06	Reference			Reference		
0.07–0.23	1.81	1.51–2.17	**<0.001**	1.65	1.24–2.20	**0.001**
≥0.24	2.01	1.68–2.41	**<0.001**	1.86	1.35–2.54	**<0.001**

*AJCC, American Joint Committee on Cancer; CI, confidence interval; HR, hazard ratio; LNR, lymph node ratio; OS, overall survival; PDAC, pancreatic ductal adenocarcinoma; TP, total pancreatectomy. Bold values meant statistically significant*.

### Construction and Validation of Nomogram

**Figure 3:**
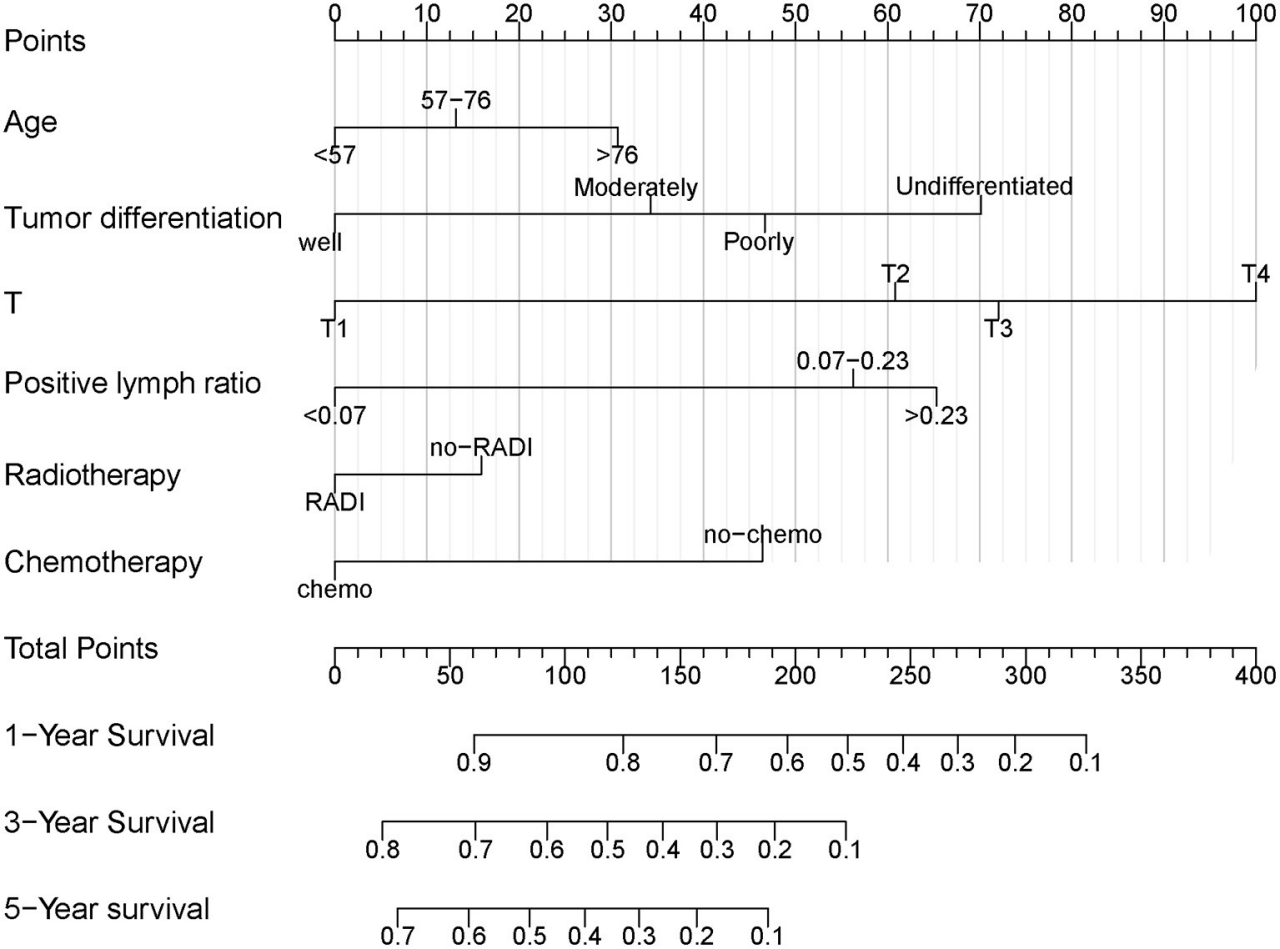
Nomogram for predicting OS of PDAC patients treated with TP. OS, overall survival; PDAC, pancreatic ductal adenocarcinoma; TP, total pancreatectomy.

Independent prognostic variables were selected for developing the nomogram for prognostic prediction of PDAC patients treated with TP. As shown in Table 3, the entire TP group was randomly divided into the training and validation cohorts. Figure 3 demonstrated the nomogram that was used for the 1-, 3-, and 5-year OS probabilities. It could be seen from the nomogram that the AJCC T stage had the greatest impact on OS. The survival probability of an individual was simply acquired by summing all scores for each factor and corresponding to the scores on the total score scale in the nomogram. Higher total scores indicated worse survival probability.

**Table 3:** Comparison of characteristics of TP patients in the training cohort and validation cohort.

**Characteristics**	**All TP patients (*n* = 1,248) *n* (%)**	**Training cohort (*n* = 873) *n* (%)**	**Validation cohort (*n* = 375) *n* (%)**	***P-*value**
Age (year)				0.98
≤ 56	255 (20.4)	179 (20.5)	76 (20.3)	
57–76	797 (63.9)	556 (63.7)	241 (64.3)	
≥77	196 (15.7)	138 (15.8)	58 (15.5)	
Gender				0.82
Female	631 (50.6)	439 (50.3)	192 (51.2)	
Male	617 (49.4)	434 (49.7)	183 (48.8)	
Marital status				0.71
Married	780 (62.5)	549 (62.9)	231 (61.6)	
Other status	468 (37.5)	324 (37.1)	144 (38.4)	
Race				0.11
White	999 (80.0)	702 (80.4)	297 (79.2)	
Black	129 (10.3)	90 (10.3)	39 (10.4)	
Asian	115 (9.2)	80 (9.2)	35 (9.3)	
Other	5 (0.4)	1 (0.1)	4 (1.1)	
Tumor location				0.46
Head	970 (77.7)	684 (78.4)	286 (76.3)	
Other	278 (22.3)	189 (21.6)	89 (23.7)	
Differentiation				0.47
Well	113 (9.1)	77 (8.8)	36 (9.6)	
Moderate	659 (52.8)	456 (52.2)	203 (54.1)	
Poor	461 (36.9)	327 (37.5)	134 (35.7)	
Undifferentiated	15 (1.2)	13 (1.5)	2 (0.5)	
Tumor size (mm)				0.75
≤ 24	297 (23.8)	213 (24.4)	84 (22.4)	
25–33	386 (30.9)	268 (30.7)	118 (31.5)	
≥34	565 (45.3)	392 (44.9)	173 (46.1)	
8th AJCC T stage				0.69
T1	129 (10.3)	96 (11.0)	33 (8.8)	
T2	697 (55.8)	482 (55.2)	215 (57.3)	
T3	367 (29.4)	256 (29.3)	111 (29.6)	
T4	55 (4.4)	39 (4.5)	16 (4.3)	
8th AJCC *N* stage				0.81
*N*0	391 (31.3)	272 (31.2)	119 (31.7)	
*N*1	522 (41.8)	362 (41.5)	160 (42.7)	
*N*2	335 (26.8)	239 (27.4)	96 (25.6)	
Chemotherapy				0.96
No	413 (33.1)	288 (33.0)	125 (33.3)	
Yes	835 (66.9)	585 (67.0)	250 (66.7)	
Radiotherapy				0.53
No	777 (62.3)	549 (62.9)	228 (60.8)	
Yes	471 (37.7)	324 (37.1)	147 (39.2)	
LNR				0.14
≤0.06	525 (42.1)	369 (42.3)	156 (41.6)	
0.07–0.23	379 (30.4)	252 (28.9)	127 (33.9)	
≥0.24	344 (27.6)	252 (28.9)	92 (24.5)	

*AJCC, American Joint Committee on Cancer; LNR, lymph node ratio; TP, total pancreatectomy*.

**Figure 4:**
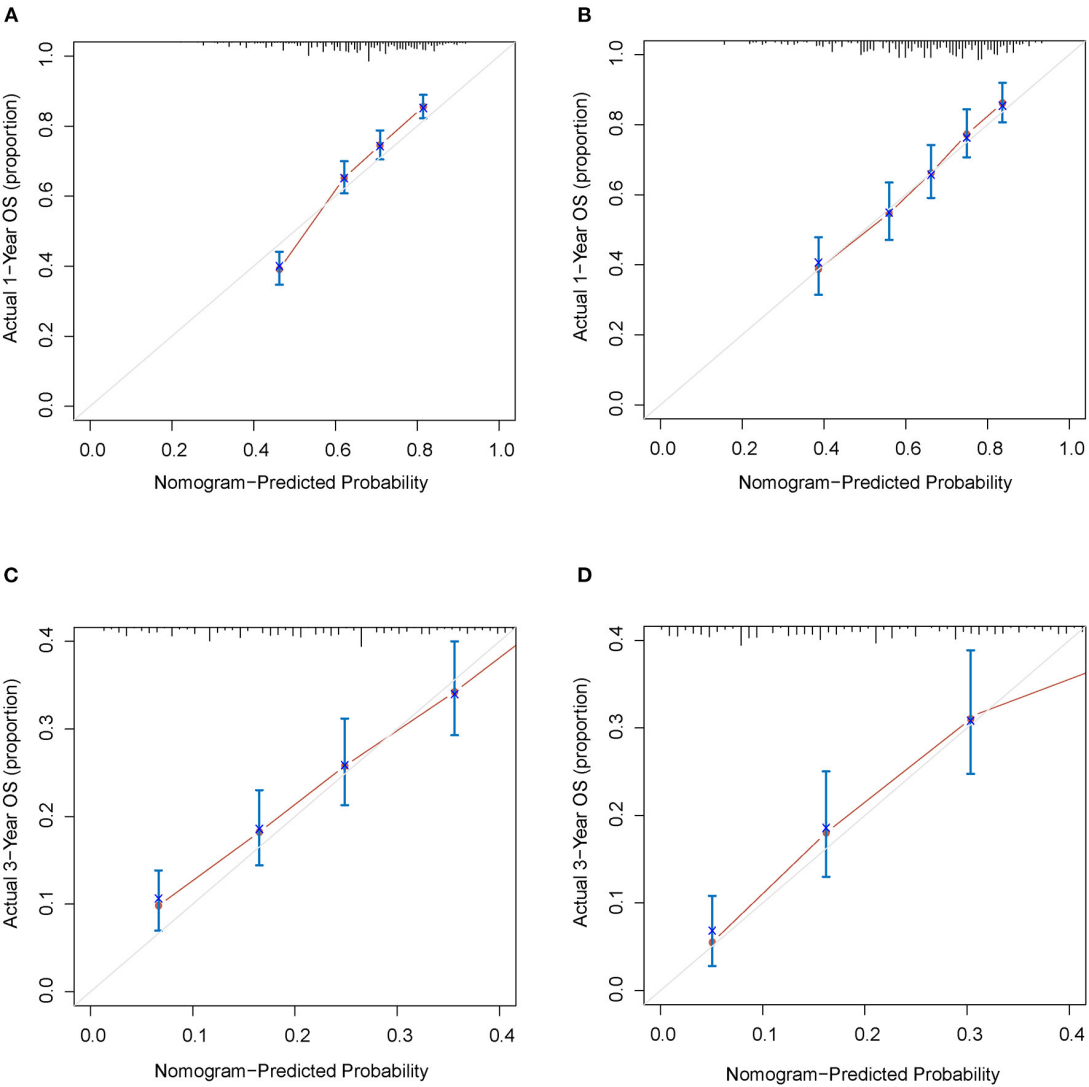
Calibration plots for 1- and 3-years OS of the nomogram. **(A)** Calibration plot of 1-year OS in the training cohort; **(B)** Calibration plot of 1-year OS in the validation cohort; **(C)** Calibration plot of 3-year OS in the training cohort; **(D)** Calibration plot of 3-year OS in the validation cohort. OS, overall survival.

**Figure 5:**
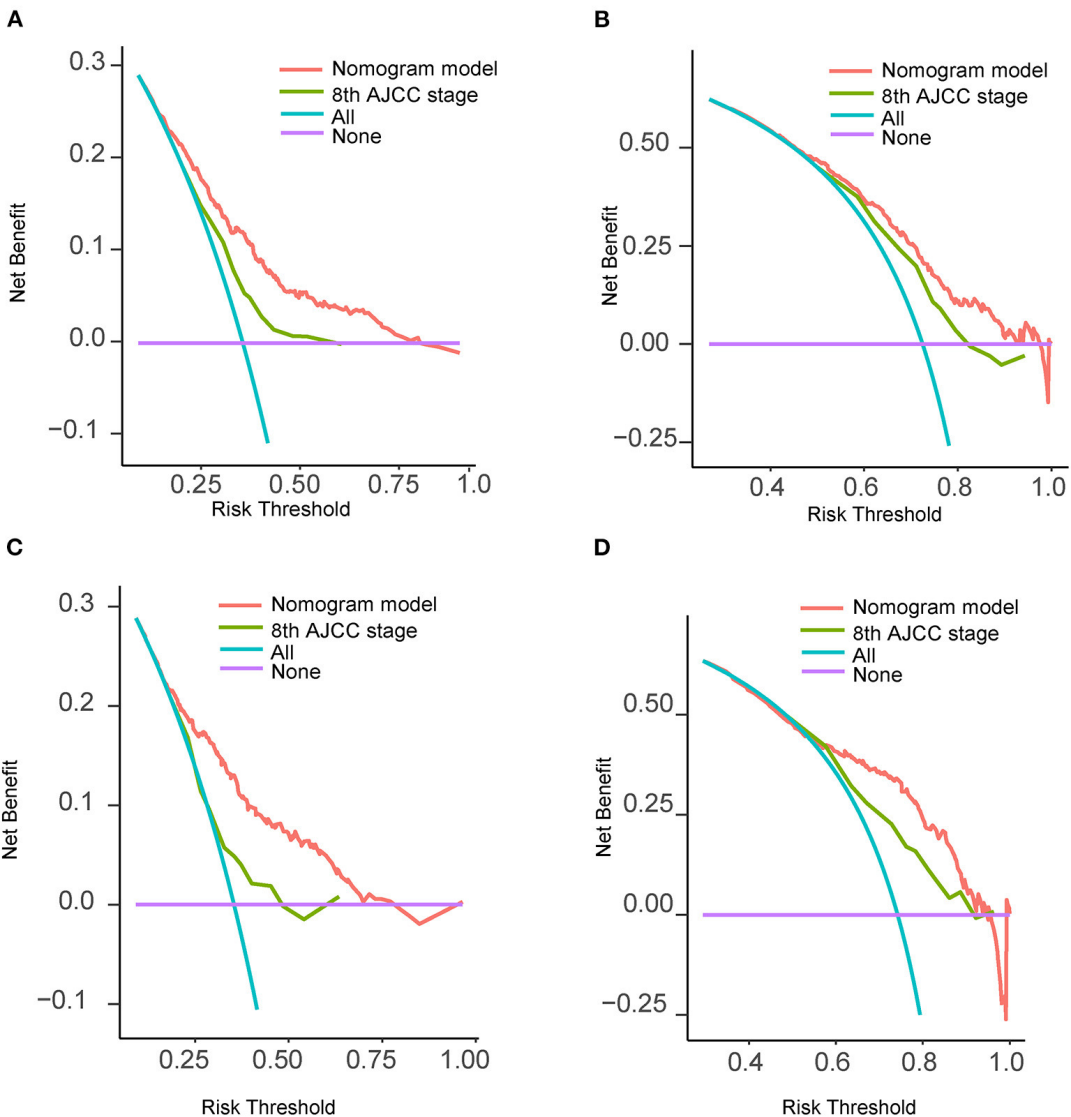
Decision curves analysis and comparison of the nomogram with the 8th AJCC TNM staging system. **(A)** 1-year OS in the training cohort; **(B)** 3-year OS in the training cohort; **(C)** 1-year OS in the validation cohort; **(D)** 3-year OS in the validation cohort. AJCC, American Joint Committee on Cancer; OS, overall survival; TNM, tumor-node-metastasis.

The C-indexes were 0.67 (95% CI: 0.66–0.68) and 0.69 (95% CI: 0.68–0.71) in the training and validation cohorts, respectively. While in the AJCC staging system, the C-indexes were 0.59 (95% CI: 0.58–0.61) and 0.60 (95% CI: 0.58–0.61) in the two cohorts, respectively. As a result, the nomogram had a more favorable discriminatory ability than the AJCC system. The predicted 1- and 3-year OS showed good unanimity with the observed situations both in the two cohorts, according to the calibration plots (Figures 4A–D). Furthermore, in both cohorts, the DCA demonstrated that the nomogram could provide satisfactory 1- and 3-year OS predictions with a preferable positive net benefit. Compared with the TNM staging system, the nomogram had better clinical practicality (Figures 5A–D).

### Risk Stratification Based on the Nomogram

**Figure 6:**
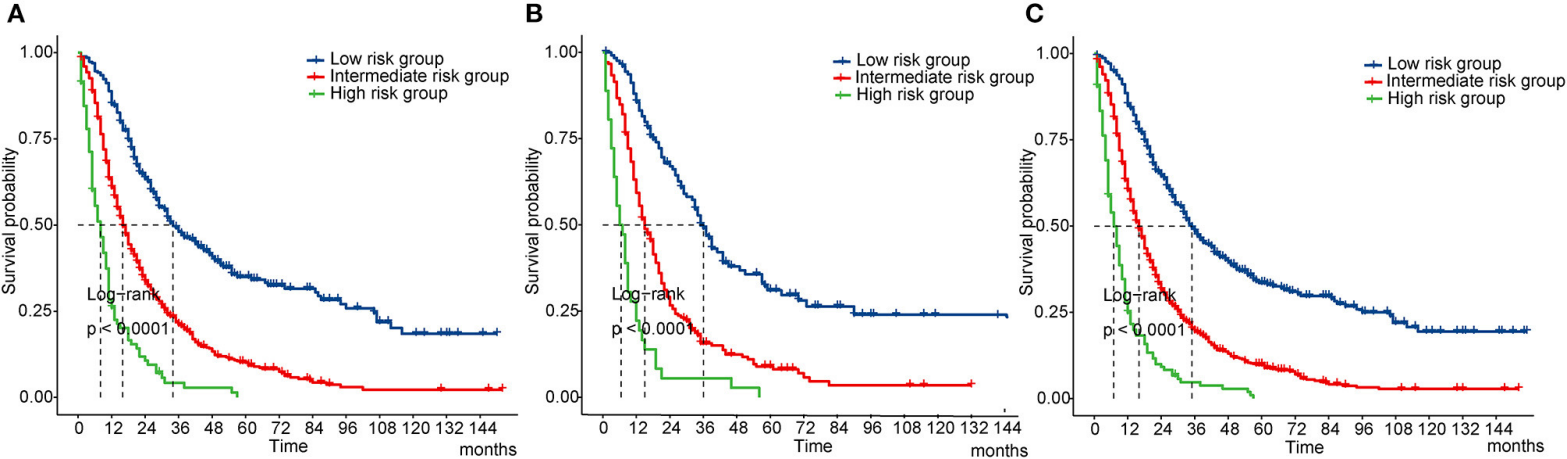
Kaplan-Meier curves of OS for risk classification based on the nomogram scores. **(A)** In the training cohort; **(B)** In the validation cohort; **(C)** In all cohort. OS, overall survival.

Finally, we performed a survival risk stratification analysis according to the cutoff values of the nomogram scores by using X-tile in the training cohort (Supplementary Figure S1D). Patients were divided into three risk groups: low-risk (total score < 123), intermediate-risk (total score: 123–217), and high-risk (total score > 217). The Kaplan-Meier curves showed significant discrimination in OS among the three groups in both cohorts and the whole cohort (Figures 6A–C).

## Discussion

In this retrospective study, based on the seer database, we conducted a PSM analysis to compare the survival of PDAC patients who were treated with TP and PD. Before and after PSM, the results consistently showed that PDAC patients following TP had similar OS and CSS compared with those following PD. Additionally, we formulated a nomogram which could effectively forecast the 1-, 3-, and 5-year OS of PDAC patients treated with TP, which might be helpful for clinicians to better grasp their patients' prognostic results. To the best of our knowledge, this is the first time that a nomogram was constructed specifically for PDAC patients treated with TP.

PDAC accounts for an overwhelming majority of pancreatic cancer, which is widely known as “the king of cancers.” Surgery plays an essential role and is considered the dominant modality in PDAC treatment. PD remains the most common surgical method for PDAC. Occasionally, PD may be inadequate to achieve complete clearance of the tumor; hence, TP may be required under this circumstance. In the 1960s and 1970s, TP reached its peak and was even regarded as a routine surgical approach for PDAC in many clinical centers (6). However, after the enthusiasm for TP, its disadvantages became obvious. Many surgeons were reluctant to choose TP in the treatment of PDAC due to increased perioperative risks and permanent pancreatic dysfunction. With the development and advances in surgical techniques, progress in researching synthetic insulin and pancreatic enzymes, TP now can be operated safely with acceptable morbidity and mortality compared with PD (26–28), and postoperative QoL has also improved (29). It was previously thought that TP was associated poorer long-term survival compared with PD (7, 14), but several studies have argued that the long-term survival of PDAC patients following TP vs. PD was equivalent (11, 12, 30). These series were almost single-centered and limited by their small sample sizes. Using data form SEER, a population-based, multi-centered and well-validated data set, the present study compared the long-term survival of PDAC patients following TP and PD. Patients with distant metastasis were excluded to remove the effects of tumor metastasis to survival. The PSM method was taken into use to minimize possible confounding effects and create well-matched cohorts. Before PSM, the results showed that OS and CSS in the unmatched TP and PD cohorts were similar. After PSM, no statistical differences in OS and CSS between the two cohorts were found. Improvement of survival in PDAC patients treated with TP may partly be due to the development of synthetic insulin, pancreatic enzyme supplementation, good glycemic control, education and self-management, which offer patients a stable postoperative metabolic status (29, 31). Therefore, the non-inferior long-term survival compared with PD may justify the use of TP for the treatment of PDAC in specific situations to achieve a complete resection, such as multifocal tumors and tumors with positive neck margins (32).

The nomogram, a simple statistical tool, has been well-recognized and widely used for prognosis prediction in which intricate mathematical models are converted to straightforward graphics (23, 33). Additionally, the nomogram can integrate various characteristics to give a more comprehensive and accurate prediction. Moreover, it can offer individualized prognosis predictions based on the characteristics of a given individual. Several studies have focused on survival prediction for patients with PDAC (34–36), but none have focus on those who are treated with TP. As mentioned above, TP can be safely performed with acceptable perioperative morbidity and mortality, and improved postoperative QoL and long-term survival. TP seems to be experiencing a renaissance in recent years; hence, it is helpful to develop a credible nomogram specifically for PDAC patients treated with TP.

Through univariate and multivariate Cox analysis, we found that age, AJCC T stage, differentiation, radiotherapy, chemotherapy and LNR were factors that significantly affected OS of the patients. Using X-tile, we obtained the optimal cutoff values of the continuous variables. Tumor characteristics were deemed to be important factors that could influence survival after pancreatic resection (37). In our model, AJCC T stage had the greatest impact on OS. The 8th AJCC system defines T4 stage as the pancreatic tumor has invaded the celiac axis, common hepatic artery, or superior mesenteric artery, which obviously leads to a poor prognosis. Tumor differentiation and age were also significantly associated with clinical outcomes, which is in agreement with previous studies (35, 36). Adjuvant chemotherapy is one element of comprehensive treatment for PDAC and is recommended in all patients (38), while radiotherapy or chemo-radiotherapy, especially in R1 resection, can be considered to improve OS of the patients (39). Our model verified that chemotherapy and radiotherapy could serve as protective factors for the patients, which proved the importance of multidisciplinary therapy (MDT) in the treatment of PDAC. The correlation between AJCC N stage and survival of the patients is controversial (40), since lymph node dissection may sometimes be insufficient. As Huebner et al. (40) reported in their study, in “N0” patients who had <11 examined lymph nodes after pancreatectomy, there was a probability that the metastatic lymph nodes were missed by harvesting too few nodes, and those patients generally had worse prognosis. We can see that under this circumstance, although the patients were judged as a favorable pathologically “N0” status, the survival turned out to be bad, which hints that *N* stage may not accurately predict survival sometimes, especially when fewer lymph nodes are moved from the patients. Riediger et al. (41) also reported that not the number of examined lymph nodes but LNR, was proved to be an independent prognostic factors after pancreas cancer resection. In this study, N stage turned out not to be a predictor in the model, whereas LNR was taken into account instead. LNR contains information on both the number of positive nodes and the total number of nodes evaluated, and increased LNR may better indicate the tendency of metastasis, as was reported in a previous study (35).

This nomogram relied on a cohort with a large sample size, which guaranteed the reliability of the results. The C-index were 0.67 (95% CI: 0.66–0.68) in the raining cohort and 0.69 (95% CI: 0.68–0.71) in the validation cohort, and calibration plots showed satisfactory consistency between the predicted and actual situations, which validated good discriminative capacity and predictive accuracy of the model. At present, the AJCC TNM system has been widely applied in clinical practice to predict the prognosis of cancer patients. However, the TNM system merely refers to the three anatomical elements of cancer but ignores other potential prognostic elements. Compared with the traditional system, our nomogram integrated more variables and demonstrated a better predictive effect. DCA puts benefit and harm together to calculate the net benefit of a prediction model, which takes clinical usefulness into consideration (25). Clinical usefulness weighs whether a prediction model can be reasonably used in clinical work, and patients can benefit from the model. In this study, the DCA curves further proved that our nomogram is superior to the TNM system with regard to clinical usefulness. Finally, based on the cutoff values of the nomogram overall scores, we formulated a risk stratification system, which could clearly differentiate patients with different survival risks.

For patients with PDAC following TP, what they concern most may be their postoperative QoL and survival time. This study successfully developed a nomogram to forecast prognosis according to the patients' clinicopathological information that could be easily obtained. Our nomogram provided a more individualized and precise prognosis prediction than the traditional AJCC staging system.

The present study had several limitations that need to be noticed. First, the study design was retrospective, which could lead to potential selection bias. Second, the SEER database lacks some important information, such as smoking and drinking status, serum CA19-9 level, surgical margin status, neurovascular invasion, detailed regimen and dosage of chemotherapy or radiotherapy, postoperative usage of insulin and pancreatic enzymes; hence we could not consider all potential prognostic factors. Third, although PSM was performed, there stilled existed some unobserved confounders, such as those mentioned above, which might affect the reliability of the results. Finally, although the nomogram and its risk classification system had been internally validated with good performance, external validation support from other independent databases or populations is still needed to further assess the model.

## Conclusions

In summary, for PDAC patients following TP, OS and CSS are similar to those who following PD. TP may be a reasonable option for PDAC patients if needed. Additionally, we developed a reliable and practical nomogram specifically for predicting the 1-, 3-, and 5-year OS of PDAC patients treated with TP, which showed superiority over the conventional AJCC staging system. This user-friendly nomogram could help clinicians make personalized survival predictions and risk assessments. Further prospective studies with more detailed clinical information and data from other large-scale cohorts are needed to improve and externally validate our model.

## Data Availability Statement

The original contributions presented in the study are included in the article/Supplementary Material, further inquiries can be directed to the corresponding author.

## Ethics Statement

Ethical review and approval was not required for the study on human participants in accordance with the local legislation and institutional requirements. Written informed consent for participation was not required for this study in accordance with the national legislation and the institutional requirements.

## Author Contributions

WS and JS: conception and design. ZL and JX: collection and assembly of data. XS, TT, and CX: data analysis and interpretation. WS: paper writing. All authors contributed to the article and approved the submitted version.

## Conflict of Interest

The authors declare that the research was conducted in the absence of any commercial or financial relationships that could be construed as a potential conflict of interest.

## Publisher's Note

All claims expressed in this article are solely those of the authors and do not necessarily represent those of their affiliated organizations, or those of the publisher, the editors and the reviewers. Any product that may be evaluated in this article, or claim that may be made by its manufacturer, is not guaranteed or endorsed by the publisher.
