# Supplementary material for: Effects of Total Pancreatectomy on Survival of Patients With Pancreatic Ductal Adenocarcinoma: A Population-Based Study
Source: Front Surg. 2021 Dec 9;8:804785. doi: 10.3389/fsurg.2021.804785 (PMC8695493; doi:10.3389/fsurg.2021.804785)

Fig. S1 optimal cutoff values of the continuous variables. **(A)** optimal cutoff values of age; **(B)** optimal cutoff values of tumor size; **(C)** optimal cutoff values of lymph node ratio; **(D)** optimal cutoff values of total nomogram scores


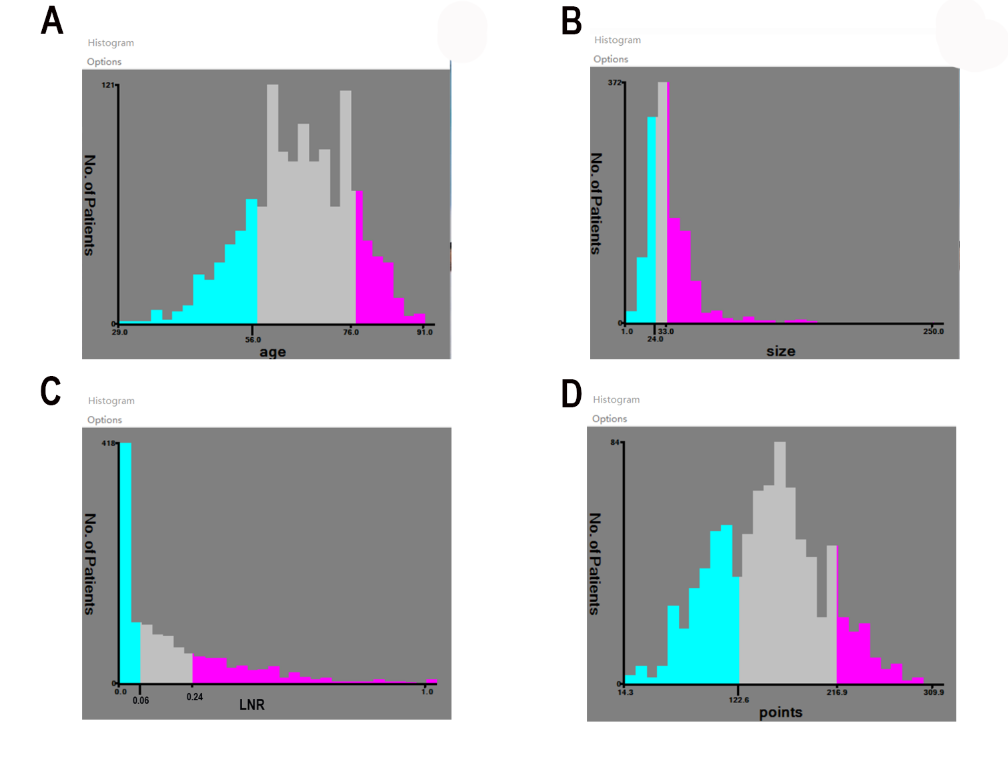

Supplement: Supplementary file 1 [file Data_Sheet_1.docx]
